# CINE-MRI to study the progress of disease in a chronic atrial fibrillation goat model

**DOI:** 10.1186/1532-429X-15-S1-E96

**Published:** 2013-01-30

**Authors:** Konrad Werys, Sathya Vijayakumar, Ravi Ranjan, Derek J Dosdall, Daniel Kim, Nassir F Marrouche, Eugene Kholmovski

**Affiliations:** 1 Cardiovascular Magnetic Resonance Group, The Cardinal Stefan Wyszynski Institute of Cardiology, Warsaw, Poland; 2 Institute of Radioelectronics, Warsaw University of Technology, Warsaw, Poland; 3 CARMA Center, Dept. of Cardiology, University of Utah, Salt Lake City, UT, USA; 4 UCAIR, Dept. of Radiology, University of Utah, Salt Lake City, UT, USA

## Background

Atrial fibrillation (AF) is a common sustained cardiac arrhythmia associated with increased risks of heart failure, left ventricle (LV) dysfunction, and known to cause an increase in left atrium (LA) volume with decrease in atrial contractility. Cine magnetic resonance images (MRI) were used to measure changes in LA and LV function in a chronic AF goat model. Ejection fraction (EF), end diastolic volume (EDV), end systolic volume (ESV), and stroke volume (SV) measurements of both the LV and LA were made from cine-MR images to track the progression of disease in goats that were induced with AF using neurostimulators.

## Methods

**Figure 1:**
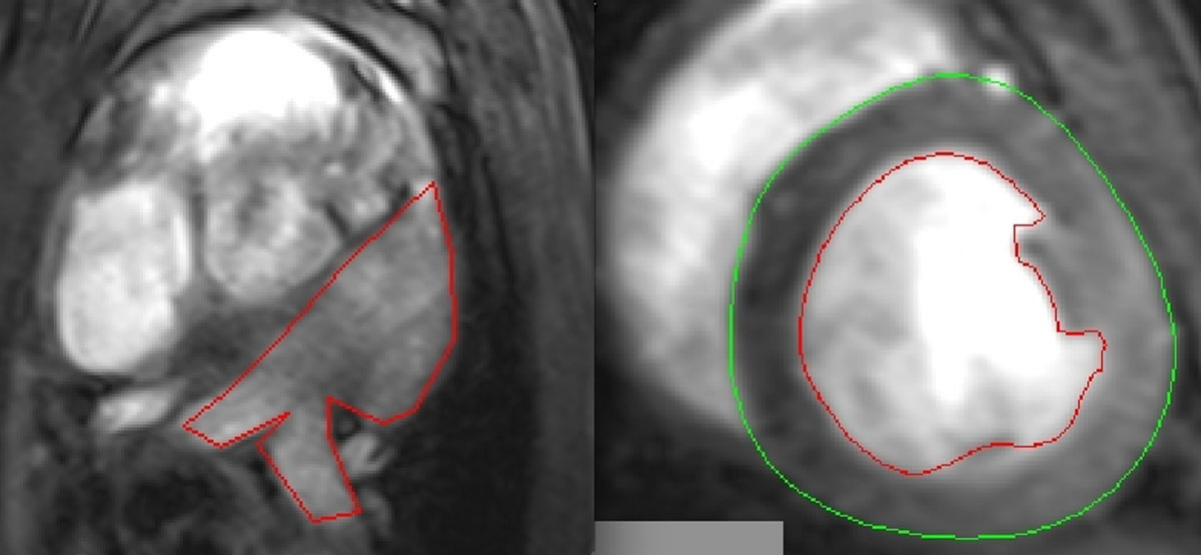
Segmentation of the LA and LV using Argus software

AF was induced in 7 goats with rapid atrial pacing. A neurostimulator (ITREL 3, Medtronic Inc, Minneapolis, MN) was implanted in the neck and delivered 50 Hz stimulation every other second at 2-3 times diastolic threshold. Prior to MRI, the device was turned off and goats that were in arrhythmia, were cardioverted to sinus rhythm with a t-wave synchronized DC shock. After MRI, the neurostimulators were turned back on. All animals underwent imaging at baseline (before device implantation) and every month afterward. Cine-MRI was performed in the short-axis view for LV evaluation and in trans-axial plane for LA evaluation. Images were acquired on a 3T Trio scanner (Siemens Healthcare, Erlangen, Germany). A 2D FLASH sequence was used in each orientation with TR/TE=3.3/1.54 ms; in-plane resolution=1.25 x 1.25 mm, slice thickness = 6 mm; flip angle=20 degrees; bandwidth = 790 Hz/pixel and zero spacing between the slices. Segmentations of the LV and LA (Figure 1) and evaluation of the EF, EDV, ESV and SV were made using the Argus software tool (Siemens Healthcare, Erlangen, Germany). Atrial EF was computed similar to the way ventricular EF is calculated ((EDV-ESV)/EDV).

## Results

**Figure 2:**
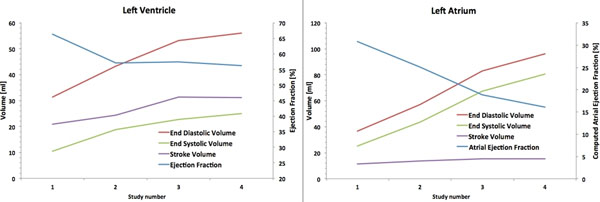
Ventricular EF, EDV, ESV, SV and computed Atrial EF, EDV, ESV and SV

Figure 2 shows the average values of EF, EDV, ESV and SV at various time points. The ventricular EF reduced by about 15% from session 1 to 4 while the computed atrial EF reduced by about 49% between session 1 and 4. Reduction in the computed atrial EF is statistically significant (p < 0.01) while that in the ventricular EF is not (p = 0.03). Significant increase in EDV and ESV were observed in both the atrium (p < 0.001) and ventricle (p < 0.001) after 3 months of atrial pacing. Drastic increase in atrial volume was also observed as seen by a factor of 2.5 increase in atrial EDV and ESV.

## Conclusions

We conclude cine-MRI is a useful tool to track the progress of disease in a chronic AF model. In the chronic AF goat model, computed atrial EF is more affected than the ventricular EF in the first 3 months after pacing (49% vs. 15%). Significant increase in LA volume was also observed following atrial pacing. Atrial pacing affects the volumes of both atrium and ventricle resulting in statistically significant increases of EDV and ESV after being paced for 3 months.

## Funding

This project was supported in part by the Ben B. and Iris M. Margolis foundation. Mr. Werys is supported by a scholarship from the European Union in the framework of European Social Fund through the Warsaw University of Technology Development Program.

